# The Potential Misdiagnosis of Multifocal Motor Neuropathy as Amyotrophic Lateral Sclerosis—A Case Series

**DOI:** 10.17925/usn.2018.14.2.102

**Published:** 2018-11-12

**Authors:** Victoria Lawson, Nathaniel M Robbins

**Affiliations:** Dartmouth Hitchcock Clinic, Geisel School of Medicine at Dartmouth, Lebanon, NH, US

**Keywords:** Amyotrophic lateral sclerosis, conduction block, differential diagnosis, multifocal motor neuropathy

## Abstract

Multifocal motor neuropathy (MMN) is a rare neuropathy that is often treatable with immunomodulatory therapy if diagnosed early. However, accurate diagnosis is difficult due to a significant overlap of symptoms with other neurological conditions, such as amyotrophic lateral sclerosis (ALS). Evidence of immunoglobulin M (IgM) anti-ganglioside GM1 antibodies and electrodiagnostic findings of conduction block are useful diagnostic criteria for MMN but are not universal findings. This review explores the differential diagnosis of MMN and ALS and discusses three cases of MMN initially diagnosed as ALS, in which the correct diagnosis allowed effective treatment. These cases highlight the need for greater awareness of MMN among physicians.

Multifocal motor neuropathy (MMN) is a rare motor neuropathy with a reported prevalence range of 0.3–3 cases in 100,000.^1^ It affects more males than females (2.7:1) and onset usually occurs before 50 years of age.^1^ The disease can progress to permanent weakness and disability but it is not life-threatening or as disabling as amyotrophic lateral sclerosis (ALS, one of the motor neuron diseases).^2^ MMN symptoms overlap with other motor-predominant diseases, such as chronic inflammatory demyelinating polyradiculoneuropathy (CIDP), ALS and ALS variants (progressive muscular atrophy, flail arm/flail leg syndromes); this overlap can introduce diagnostic uncertainty.

Recognizing the significant overlap in the presentation of MMN and ALS is particularly important given that MMN is treatable, whereas ALS is rapidly fatal and non-treatable. The profound psychosocial impact of an ALS diagnosis is well established and includes grief, depression, anxiety, feelings of hopelessness and other negative, life-altering psychological effects.^3,4^ While MMN can be debilitating, it does not carry the same grave prognosis as ALS and offers the hope of treatment options.

ALS inexorably progresses, spreading to involve multiple different motor functions before eventually resulting in death. The median survival time is 3–5 years and only around 10% survive to 10 years.^5,6^ The only disease-modifying treatment options for ALS are riluzole and edaravone.^7–12^ In contrast, MMN is associated with a normal life expectancy and has several treatment options available,^13–15^ though MMN can result in progressive muscle weakness that may lead to severe disability if left untreated.^16^ Early treatment with intravenous immunoglobulin (IVIg) is essential to ensure optimal treatment response and prevent progression to axonal loss.^17^ It is critical therefore that MMN is correctly diagnosed as early as possible, enabling commencement of appropriate therapy to prevent permanent effects, reduce disability, and avoid the psychological distress of being misdiagnosed with ALS—a uniformly fatal illness. Unfortunately, MMN can be very difficult to diagnose in certain cases, particularly early in the disease course, and in the absence of obvious conduction block (CB) and GM1 antibodies.

This article aims to discuss the differential diagnosis of MMN and ALS, through a series of illustrative case studies.

## Clinical features of multifocal motor neuropathy and amyotrophic lateral sclerosis

ALS is insidious in onset and affects more men than women.^1^ Most people who develop the disease are aged between 40 and 70, with an average age of 55 at the time of diagnosis.^18^ However, ALS also occurs in people in their twenties and thirties.^1^ Onset is asymmetric, with weakness developing in a focal region of the face, arm or leg. Patients show signs of spasticity, rapid muscle atrophy, weakening and wasting. This leads to an inability to walk or move the arms. Muscle weakening progresses to the chest muscles, ultimately leading to respiratory failure. Like MMN, ALS can present with a foot drop or distal upper extremity weakness and atrophy, although it is more likely to involve a portion of a limb rather than a single nerve distribution. Like in ALS, patients with MMN can manifest muscle atrophy and fasciculations, although fasciculations are more prominent in ALS (Table 1).^1,19^

While early diagnosis of MMN can be difficult in atypical cases, there are cardinal clinical features that can help establish the diagnosis. MMN is characterized by asymmetric limb weakness without sensory loss, more commonly affecting the upper extremities. The weakness is patchy and multifocal, corresponding to the distribution of single nerves rather than segmental or radicular (Table 1).^1,20,21^ Like ALS, the disease has a progressive course, but progression of weakness tends to be stepwise, rather than insidious. Core clinical criteria require involvement of at least two separate motor nerves without objective sensory abnormalities, except for mild vibratory sense impairment. A characteristic electrophysiological pattern is focal slowing and CB of motor nerve fibers within nerve segments. Cervical nerve root stimulation is also an important technique for assessing CB since approximately 13% of all CBs in MMN are proximal and may be missed with routine nerve conduction studies.^22,23^

Unlike ALS, which is believed to be caused by a combination of genetic and environmental factors, MMN clearly has an autoimmune etiology; it is associated with elevated anti-GM1 IgM levels in ~50% of patients and responds to immunomodulatory treatment.^21,24–26^ The pathophysiology of MMN and CB has been covered elsewhere and will not be reviewed here.^24^

## Diagnosis of multifocal motor neuropathy and amyotrophic lateral sclerosis

A definitive diagnosis of MMN is based on core clinical criteria set out by the European Federation of Neurological Societies (EFNS)/Peripheral Nerve Society (PNS) (core, supportive and exclusion criteria in MMN diagnosis are defined by the EFNS; Table 2).^28^ Core criteria include slowly progressive or stepwise progressive, focal, asymmetric limb weakness, in the distribution of at least two nerves, for more than one month; and no objective sensory abnormalities, except for minor vibratory abnormalities in the lower limbs.

Important features that would exclude a diagnosis of MMN include sensory loss or sensory symptoms other than mild vibratory loss in the toes, or slight paresthesia; as well as symmetric weakness at onset.^29^ However, these would also be atypical for ALS. In terms of differentiation from ALS and other motor-predominant neuropathic disorders, other exclusion criteria for MMN that would help distinguish it from ALS include upper motor neuron signs and bulbar weakness. IgM anti-ganglioside GM1 antibodies are present in approximately half of patients with MMN (30–80% depending on series).^30,31^ However, they have also been associated with other immune-mediated neuropathies, non-immune-related neuropathies and even patients with ALS.^32–34^ Therefore, anti-GM1 antibodies are diagnostically helpful but cannot be relied upon absolutely.

The hallmark finding of motor CB has also been left out of the EFNS diagnostic criteria, as CB in patients with otherwise typical MMN may not be detectable using standard clinical electrophysiologic testing, a test that requires considerable electrophysiological expertise. CB is defined as the failure of action potential propagation at a given site along a single axon and its presence outside the usual sites of nerve compression on nerve conduction testing constitutes the hallmark of MMN. The EFNS criteria allow for definite and probable CB.^28^ Definite CB is defined as follows: “Negative peak compound muscle action potential (CMAP) area reduction on proximal versus distal stimulation of at least 50% whatever the nerve segment length (median, ulnar, and peroneal). Negative peak CMAP amplitude on stimulation of the distal part of the segment with motor CB must be >20% of the lower limit of normal and >1 mV and increase of proximal to distal negative peak CMAP duration must be ≤30%”. Probable CB is defined by: “negative peak CMAP area reduction of at least 30% over a long segment (e.g., wrist to elbow or elbow to axilla) of an upper limb nerve with increase of proximal to distal negative peak CMAP duration ≤30%” or “Negative peak CMAP area reduction of at least 50% (same as definite) with an increase of proximal to distal negative peak CMAP duration >30%”.^28^ Unlike axonal loss, CB will be undetectable if located at a site that is not accessible by conventional nerve conduction testing. Proximal root stimulation should be considered in these cases.^22,23^ In one study of patients with pure lower motor neuron disease without motor CB, 10% responded to IVIg therapy.^35^ Therefore, the absence of CB or GM1 antibodies argues against IVIg responsiveness, but does not rule it out.

In addition to CB and other demyelinating abnormalities, there may also be evidence of motor axon loss, such as decreased distally evoked CMAP and signs of denervation and re-innervation on needle electromyography.^36^ Magnetic resonance neurography is another useful diagnostic technique; focal enlargement and increased signal intensity of the brachial plexus is seen on T2-weighted images in MMN.^37^ In addition, many centers are using ultrasound to detect nerve enlargement; in a recent study, high-resolution sonography of peripheral nerves revealed distinct multifocal nerve enlargement patterns, which may support a diagnosis of MMN. Ultrasound findings did not correlate well with clinical severity or electrophysiological findings at initial presentation, but changes in the Ultrasound Pattern Sum Score (UPSS) correlated well with the clinical course in terms of muscle strength, as measured by the Medical Research Council sum score.^38^ Ultrasound has been used to differentiate MMN from other neuropathies^39^ and may also be a useful tool for therapeutic monitoring.

Diagnosis of ALS employs the El Escorial criteria and Awaji algorithm.^40^ According to these criteria, diagnosis of ALS requires signs of lower motor neuron degeneration by clinical, electrophysiological or neuropathologic examination, and signs of upper motor neuron degeneration by clinical examination. In addition, there must be evidence of progressive spread of signs within a region or to other regions, absence of electrophysiological evidence of other disease processes, and absence of neuroimaging evidence of other disease processes that might explain the observed clinical and electrophysiological signs.

The significant clinical overlap between these two motor processes means that misdiagnosis is common and can be difficult to avoid. It is important to identify features that help distinguish MMN from ALS. Some of these differences have been mentioned previously but deserve further emphasis. While any body part can be affected in ALS, MMN almost always presents with wrist drop, or less frequently, foot drop.^17^ As such, MMN presents in a “patchy” distribution, while ALS involves a segment such as a limb and spreads insidiously rather than in a stepwise manner. Bulbar and respiratory involvement are rarely seen in MMN but are often present in ALS. The muscle weakness associated with MMN involves less atrophy, except in severe cases or in individuals who have had the disease for many years.^41^ Fasciculations occur in both MMN and ALS but are more prominent and widespread in ALS. Additionally, in ALS, fasciculations are not necessarily restricted to weak muscles.^1,19^ The absence of upper motor neuron signs is probably one of the most important clinical indicators of MMN over ALS. However, this finding must also be interpreted with caution since there are lower motor neuron ALS variants that are not uncommon and also lack upper motor neuron signs (e.g., progressive muscular atrophy). Electrophysiologically, MMN is distinguished from ALS by CB, and by extension, reduced recruitment of motor units in the absence of axonal injury. Reduced recruitment is also seen in ALS but is generally accompanied by more prominent denervation on needle electromyography.^1,42^

Diagnosis of MMN or ALS can take more than a year, and a diagnostic delay is associated with worse prognosis. In a US study of 46 patients with MMN referred to a tertiary neuromuscular center, only 6 were previously given the correct diagnosis.^2^ The ratio of MMN to ALS is approximately 1 to 20, and patients with MMN are often diagnosed as having ALS.^41,43^ The correct diagnosis of MMN often requires the involvement of a neuromuscular specialist with sufficient expertise.

## Treatments for multifocal motor neuropathy and evidence for their use

Current EFNS/PNS guidelines recommend IVIg as the standard, evidence-based therapy for MMN.^44^ Good response to IVIg is seen in up to 80% of patients with MMN.^21,45^ Subcutaneous immunoglobulin administration has also shown efficacy in MMN and is more convenient than intravenous administration.^46–48^ Further developments such as hyaluronidase-facilitated administration and concentrated formulations may facilitate subcutaneous administration.^49^

Various other treatments including cyclophosphamide, rituximab, mycophenolate mofetil, beta-interferon, cyclosporine, azathioprine, and infliximab have all been used to treat MMN, but insufficient clinical trial data support their use for this indication.^50^ Plasma exchange or corticosteroids are ineffective or harmful in MMN, and their use should be avoided.^51,52^ High-dose cyclosporins have also shown some efficacy but have toxicity issues, and data supporting their use are limited.^53^ Rituximab has shown some efficacy in patients with MMN, but data are mixed and need confirmation in a large clinical trial.^54^

Treatment options for ALS are more limited. Currently two drugs are approved that delay the progression of ALS: the anti-excitotoxic drug riluzole, which has been available for over 20 years,^8,9^ and the recently approved edaravone, an antioxidant.^55^ However, edaravone has demonstrated efficacy only in a subset of patients with early stage ALS who meet specific criteria (ALS of grade 1 or 2 in the Japan ALS Severity Classification, scores of at least 2 points on all 12 items of the Revised ALS Functional Rating Scale [ALSFRS-R], forced vital capacity of 80% or more, definite or probable ALS according to the revised El Escorial criteria, and disease duration of 2 years or less).^56^

## Case studies

The following series of cases are taken from the authors’ experience and are typical of the challenges in differentiating MMN from ALS (Table 3).

### Case 1

A 61-year-old male presented 7 years previously with painless weakness of the right hand following elbow surgery. Based on the presence of weakness and atrophy, a clinical diagnosis of ulnar neuropathy was made. At re-assessment 2 years later, he had developed recurrence of right hand weakness without sensory disturbance. Although without numbness or paresthesia, he did report mild discomfort localized to the shoulder girdle in the subscapular region extending to the rhomboids and involving the proximal arm. The discomfort subsided quickly, giving way to more profound atrophy of the hand and forearm. Injury to the posterior interosseous nerve was suspected based on finger and wrist extension weakness, without associated elbow extension weakness or sensory alteration on examination.

An electrodiagnostic study performed at an outside hospital suggested injury to the radial nerve and ulnar neuropathy at the elbow; CB was not identified. Other limbs were not studied. Initial orthopedic impression was of a post-traumatic radial neuropathy related to an elbow injury during martial arts training. The patient was referred for neurologic and repeat electrodiagnostic studies. Nerve conduction studies revealed low amplitude radial, ulnar, and median motor responses on the right, with preserved sensory conduction, but did not fulfill the criteria for CB. Needle electromyography revealed prominent fibrillation potentials and positive sharp waves in distal muscles of the right hand; less prominent denervation (tr–1+) in biceps and triceps muscles with associated neurogenic recruitment abnormalities; sparse fibrillation potentials and positive sharp waves in the deltoid and extensor digitorum communis of the left upper extremity; and complex repetitive discharges in the medial gastrocnemius muscles without evidence for active denervation or chronic motor unit changes. A disorder of motor neurons or their axons was suspected based on these findings.

ALS was the leading diagnosis due to the absence of definite CB; presence of active denervation in multiple myotomes of the right upper extremity and less prominently the left upper extremity; and more subtle neurogenic changes in the medial gastrocnemius muscles of the lower extremities. Evidence against ALS included the absence of upper motor neuron signs, the absence of evidence for chronic denervation and re-innervation, and lack of significant progression over 2–3 years.

Ancillary serologic testing revealed a mildly elevated rheumatoid factor and serum creatine kinase. Cerebrospinal fluid (CSF) protein was also mildly elevated. Differential diagnosis included MMN, Lewis Sumner variant of CIDP, and ALS or another motor neuron variant.

A course of immunoglobulin was initiated at 3-week intervals after a loading dose of 2 g/kg over 5 days. This resulted in clinical improvement with increased strength of the right upper extremity and gradual restoration of muscle bulk in the right hand.

This case highlights some important difficulties in distinguishing MMN from motor neuron disease. In particular, the absence of definite CB can make the diagnosis essentially indistinguishable from a lower motor neuron variant of ALS. The detection of CB requires technical proficiency including attention to distance measurements, supramaximal stimulation, and optimal placement of electrodes. Even with technical proficiency, CB may not fulfill criteria deemed “definite”, or may be proximal to recording sites. This underscores the importance of needle electromyography as part of the electrophysiologic diagnosis. In particular, recruitment abnormalities without active (fibrillation potentials and positive sharp waves) or chronic denervation may be an important clue.

The absence of significant progression is an additional important aspect of diagnosis; ALS inevitably progresses, and the diagnosis is confirmed if a patient develops defining features such as bulbar dysfunction or upper motor neuron signs.

### Case 2

The patient is a 70-year-old male diagnosed with ALS after developing bilateral hand weakness that left him unable to work as a sculptor. His initial complaints were of weakness of the right hand, as manifest by loss of dexterity then rapid loss of muscle bulk in his right hand and forearm. This was followed by weakness of the right lower extremity with foot drop. Left distal lower extremity weakness followed but remained milder than on the right. CB was not detected on initial electrodiagnostic study; only two motor nerves were studied (ulnar and median nerve on the right). Because of these symptoms and the absence of CB on electrodiagnostic study, he was given a diagnosis of ALS.

The patient was referred for another opinion when he failed to progress as was expected for his diagnosis of ALS. At the time of his re-evaluation, three of his four limbs were affected. Weakness most prominently affected his finger extensors, hand intrinsics, and thumb abduction on the right. Affected muscle groups were wasted, but there was no sensory loss. His speech was clear without dysarthria or dysphonia. Tongue was normal without fasciculations. Muscle stretch reflexes were depressed throughout. The presence of muscle cramping, most prominently in the legs and trunk, prompted magnetic resonance imaging of the thoracic cord, which was unrevealing for spinal cord or root enhancement. Serologic studies revealed elevated creatine kinase on two separate occasions. Ganglioside titers were negative for GM1 antibody. The diagnosis of MMN was confirmed by the finding of CB in non-compressive locations in the right ulnar, radial, and peroneal nerve. There was some active denervation in the anterior tibialis and medial gastrocnemius but this was modest and was not accompanied by evidence of significant motor unit changes. Other muscles studied were normal with the exception of pronounced recruitment abnormalities in radial- and ulnar-innervated muscles without evidence for active denervation.

IVIg was initiated and resulted in improvement of weakness in all extremities. Right upper extremity strength improved but did not return to baseline. An immunoglobulin (Ig) dependency assessment was performed ~8 months after initiation of regular IVIg and he had deteriorated with respect to strength in both upper and lower extremities with recurrence of left lower extremity foot drop. Regularly scheduled IVIg was resumed and resulted in a return to the strength observed prior to Ig dependency test.

This case highlights the role of re-evaluation in the assessment of patients with atypical ALS. Re-evaluation was prompted when the patient did not progress as expected for his disease. Features that were atypical included the absence of bulbar symptoms and the absence of upper motor neuron signs with diffusely depressed reflexes.

On re-evaluation, the patient had progressed to involve multiple motor nerves, and evidence of CB in the ulnar and radial motor nerves cinched the diagnosis. The presence of multiple mononeuropathies can mimic the myotomal distribution of ALS at presentation. This also underscores the importance of maintaining an index of suspicion early in the course of the illness to detect a stepwise pattern of weakness suggestive of a multifocal mononeuropathy rather than a myotomal distribution of weakness. The absence of denervation on needle electromyography should be an important diagnostic clue for an alternate diagnosis, and the disproportionate recruitment abnormalities fits with MMN. It should be noted that in MMN, proximal CB may not be detectable by nerve conduction studies but may be appreciated by recruitment abnormalities. Additionally, this case underscores the importance of adequate sampling of motor nerves early with attention to performing proximal stimulations.

### Case 3

A 52 year-old-male presented to the orthopedic clinic with complaints of “his right ring finger getting stuck” after using shears for a prolonged period. His weakness had progressed over the course of the preceding 12–18 months. In reviewing his history, he admitted that the progression was “intermittent”, reporting abrupt worsening of hand strength in the 4–6 months prior to presentation. He had difficulties with flexion of the 4^th^ and 5^th^ digits of the right hand and he held his 5^th^ digit in an abducted position. He could not hold his 5^th^ digit in full extension and had weakness of abduction of the fingers. There was no associated pain, numbness or tingling but he did complain of cramping localized to the arms, upper back and shoulders.

He was referred for neurologic consultation and electrodiagnostic evaluation because of hypothenar atrophy; a putative diagnosis of ulnar neuropathy was suggested. Neurologic examination revealed normal cranial nerves without evidence of bulbar dysfunction, tongue atrophy or fasciculations. On motor examination, he had mild atrophy of muscles of the right thenar, hypothenar and forearm. He had mild left thenar atrophy. Strength testing revealed 4-/5 of right finger abduction and 5-/5 in finger extension; 4-/5 left thumb abduction; and 4/5 right ankle and toe dorsiflexion. Shoulder abduction, elbow flexion and elbow extension strength were normal bilaterally, as was proximal lower extremity strength. Reflexes were 3+ at the patella, absent at the right ankle, 2+ at the left ankle, and decreased in the bilateral upper extremities. The electrodiagnostic study failed to identify CB but the right median motor response amplitude was reduced. Denervation was present in the first dorsal interosseous but was not prominent. There were no motor unit changes such as polyphasia, large amplitude or long duration units.

Ganglioside antibodies revealed GM1 positivity at a high titer. CSF evaluation was negative for elevated protein or cells. Based on the presence of prominent recruitment abnormalities, GM1 positivity and a history of stepwise change in hand function, a trial of IVIg was completed. A repeat neurologic evaluation ~6 months after Ig therapy revealed improvement in the strength of thumb abduction on the left and ankle dorsiflexion on the right. Right finger abduction was also improved but to a lesser degree. A diagnosis of MMN was suspected based on this response and he remained on booster IVIg.

## Future perspectives

As illustrated by these case examples, there is a need for further techniques to distinguish ALS from MMN. The two conditions have been found to exhibit distinct cytokine and chemokine profiles in patients. A 2015 study (n = 56) found differences in CSF inflammatory features between patients with MMN and those with ALS; in particular, fibroblast growth factor-2 and granulocyte colony-stimulating factor levels were elevated in patients with ALS compared with those with MMN.^57^

In addition, as discussed earlier, recent data has shown that nerve ultrasound has high diagnostic accuracy in the differential diagnosis of ALS and MMN, and might be superior to nerve conduction studies in the diagnosis of MMN in hospitalized patients with this differential diagnosis.^38,58^ Another recent study suggested that cervical root sonography may be a useful technique to support the diagnosis of MMN rather than ALS, even in the absence of CB.^59^ A more rapid and accurate diagnosis may lead to greater numbers of patients receiving early IVIg or other MMN treatments, though the long-term outcome of more widespread early diagnosis remains unknown.

## Summary and concluding remarks

The misdiagnosis of MMN as ALS is an important issue with serious clinical implications for the patient due to differences in prognosis and treatment. In this review, some important clinical features were discussed that may help to distinguish the two disorders. In summary, the following features should alert the physician to a possible diagnosis of MMN: (i) distal upper limb involvement (although the physician should also be aware that this is a common presenting symptom of ALS); (ii) multifocal, stepwise progression in the distribution of single nerves; (iii) absence of bulbar/respiratory involvement; (iv) CB/demyelinating abnormalities on electrophysiologic study; (v) absence of upper motor neuron signs; (vi) sparse fasciculations and (vii) GM1 antibodies.^1,20,21,29^

However, it bears repeating that ALS variants without upper motor neuron signs can be exceedingly difficult to distinguish from immune-responsive lower motor neuron syndromes such as MMN. The presence of GM1 antibodies or CB should always prompt consideration of an IVIg trial; given that most patients will respond by 8–12 weeks of booster doses, this is appropriate given the gravity of a misdiagnosis. Similarly, in patients with pure lower motor neuron syndromes without CB or GM1 antibodies, a trial of IVIg should be considered.

There is a need for dissemination of information about the clinical diagnosis of MMN. The above presentations of ‘real world’ cases demonstrate how misdiagnosis can be avoided. Important considerations are the need for technical expertise in electrodiagnostic testing, since definite CB is not always present, and the need for repeat neurologic evaluations. When correctly diagnosed, MMN often responds well to IVIg. However, delayed diagnosis will impair treatment effectiveness and can result in decreased muscle strength, disability, and poorer prognosis. ❐

## Figures and Tables

**Table 1:** Characteristic features and treatment of multifocal motor neuropathy and amyotrophic lateral sclerosis

	MMN	ALS
Clinical features	Asymmetric, distal > proximal, upper limb > lower limb weakness without sensory loss. Some patients with subjective sensory loss, pain, and fatigue Decreased tendon reflexes	Asymmetric weakness without sensory loss May have upper motor neuron signs and cognitive involvement, usually more prominent muscle atrophy Increased tendon reflexes
Laboratory features	CSF protein usually normal or slightly elevated 40–50% of patients may have IgM ganglioside antibodies	CSF protein usually normal or slightly elevated No significant titers of IgM ganglioside antibodies
Electrodiagnostic findings	Multifocal demyelinating motor neuropathy with or without conduction block	No focal demyelinating lesions. Active and chronic motor axon loss and fasciculations in multiple regions
Treatment	IVIg, rituximab, and cyclophosphamide Does not respond to steroids or plasma exchange	Supportive treatment

ALS = amyotrophic lateral sclerosis; CSF = cerebrospinal fluid; IgM = immunoglobulin M; IVIg = intravenous immunoglobulin; MMN = multifocal motor neuropathy.

Adapted from Lawson et al., 2014.^1^

**Table 2:** Clinical criteria required for the diagnosis of multifocal motor neuropathy

Core criteria	Supportive criteria	Exclusion criteria
• Slowly progressive or stepwise progressive, focal, asymmetric limb weakness; i.e., motor involvement in the motor nerve distribution of at least two nerves for more than 1 month. If symptoms and signs are present only in the distribution of one nerve, only a possible diagnosis can be made • No objective sensory abnormalities except for minor vibration sense abnormalities in the lower limbs	• Predominant upper limb involvement • Decreased or absent tendon reflexes in the affected limb • Absence of cranial nerve involvement • Cramps and fasciculations in the affected limb • Response in terms of disability or muscle strength to immunomodulatory therapy	• Upper motor neuron signs • Marked bulbar involvement • Sensory impairment more marked than minor vibration loss in the lower limbs • Diffuse symmetric weakness during the initial weeks

Adapted with permission from European Federation of Neurological Societies and the Peripheral Nerve Society, 2010.^28^

**Table 3:** Summary of clinical cases

	Nerve conduction studies	Laboratory tests	Ganglioside antibody presence	Initial diagnosis
Case 1	No CB, evidence of denervation in upper extremities, subtle changes in lower extremities	Rheumatoid factor, CSF protein and CK were all mildly elevated	Not tested	Differential diagnosis included MMN, Lewis Sumner variant of CIDP, ALS and motor neuron variant
Case 2	No CB in ulnar and median nerve (CB was detected later)	CK elevated	GM1 negative	ALS
Case 3	No CB (reduced median motor amplitude)	CSF evaluation negative for elevated protein or cells	GM1 positive	Differential diagnosis included MMN, ALS, distal hereditary motor neuropathy

ALS = amyotrophic lateral sclerosis; CB = conduction block; CIDP = chronic inflammatory demyelinating polyradiculoneuropathy; CK = creatine kinase; CSF = cerebrospinal fluid; MMN = multifocal motor neuropathy.

## References

[1] Lawson VH, Arnold WD. Multifocal motor neuropathy: a review of pathogenesis, diagnosis, and treatment. Neuropsychiatr Dis Treat. 2014;10:567–76. 24741315 10.2147/NDT.S39592 PMC3983019

[2] Taylor BV, Wright RA, Harper CM, Natural history of 46 patients with multifocal motor neuropathy with conduction block. Muscle Nerve. 2000;23:900–8. 10842266 10.1002/(sici)1097-4598(200006)23:6<900::aid-mus9>3.0.co;2-y

[3] Pagnini F, Psychological wellbeing and quality of life in amyotrophic lateral sclerosis: a review. Int J Psychol. 2013;48:194–205. 22731673 10.1080/00207594.2012.691977

[4] Mitchell JD, Borasio GD. Amyotrophic lateral sclerosis. Lancet. 2007;369:2031–41. 17574095 10.1016/S0140-6736(07)60944-1

[5] Zarei S, Carr K, Reiley L, A comprehensive review of amyotrophic lateral sclerosis. Surg Neurol Int. 2015;6:171. 26629397 10.4103/2152-7806.169561 PMC4653353

[6] Pupillo E, Messina P, Logroscino G, Long-term survival in amyotrophic lateral sclerosis: a population-based study. Ann Neurol. 2014;75:287–97. 24382602 10.1002/ana.24096

[7] Zoccolella S, Beghi E, Palagano G, Riluzole and amyotrophic lateral sclerosis survival: a population-based study in southern Italy. Eur J Neurol. 2007;14:262–8. 17355545 10.1111/j.1468-1331.2006.01575.x

[8] Bensimon G, Lacomblez L, Meininger V. A controlled trial of riluzole in amyotrophic lateral sclerosis. ALS/Riluzole Study Group. N Engl J Med. 1994;330:585–91. 8302340 10.1056/NEJM199403033300901

[9] Lacomblez L, Bensimon G, Leigh PN, Dose-ranging study of riluzole in amyotrophic lateral sclerosis. Amyotrophic Lateral Sclerosis/Riluzole Study Group II. Lancet. 1996;347:1425–31. 8676624 10.1016/s0140-6736(96)91680-3

[10] Miller RG, Mitchell JD, Moore DH. Riluzole for amyotrophic lateral sclerosis (ALS)/motor neuron disease (MND). Cochrane Database Syst Rev. 2012;CD001447. 11687111 10.1002/14651858.CD001447

[11] Cruz MP. Edaravone (Radicava): a novel neuroprotective agent for the treatment of amyotrophic lateral sclerosis. P T. 2018;43:25–8. 29290672 PMC5737249

[12] Writing Group; Edaravone (MCI-186) ALS 19 Study Group. Safety and efficacy of edaravone in well defined patients with amyotrophic lateral sclerosis: a randomised, double-blind, placebo-controlled trial. Lancet Neurol. 2017;16:505–12. 28522181 10.1016/S1474-4422(17)30115-1

[13] Azulay JP, Blin O, Pouget J, Intravenous immunoglobulin treatment in patients with motor neuron syndromes associated with anti-GM1 antibodies: a double-blind, placebo-controlled study. Neurology. 1994;44:429–32. 8145910 10.1212/wnl.44.3_part_1.429

[14] Leger JM, Chassande B, Musset L, Intravenous immunoglobulin therapy in multifocal motor neuropathy: a double-blind, placebo-controlled study. Brain. 2001;124:145–53. 11133794 10.1093/brain/124.1.145

[15] Leger JM, Vargas S, Lievens I. Efficacy of intravenous immunoglobulin in multifocal motor neuropathy. Ann N Y Acad Sci. 2007;1110:248–55. 17911439 10.1196/annals.1423.026

[16] Lange DJ, Weimer LH, Trojaborg W, Multifocal motor neuropathy with conduction block: slow but not benign. Arch Neurol. 2006;63:1778–81. 17172619 10.1001/archneur.63.12.1778

[17] Cats EA, van der Pol WL, Piepers S, Correlates of outcome and response to IVIg in 88 patients with multifocal motor neuropathy. Neurology. 2010;75:818–25. 20805527 10.1212/WNL.0b013e3181f0738e

[18] The ALS Association. Who Gets ALS? Available at: www.alsa.org/about-als/facts-you-should-know.html (accessed October 17, 2018).

[19] de Carvalho M, Kiernan MC, Swash M. Fasciculation in amyotrophic lateral sclerosis: origin and pathophysiological relevance. J Neurol Neurosurg Psychiatry. 2017;88:773–9. 28490504 10.1136/jnnp-2017-315574

[20] Leger JM, Guimaraes-Costa R, Iancu Ferfoglia R. The pathogenesis of multifocal motor neuropathy and an update on current management options. Ther Adv Neurol Disord. 2015;8:109–22. 25941538 10.1177/1756285615575269 PMC4409549

[21] Vucic S, Black KR, Chong PS, Multifocal motor neuropathy: decrease in conduction blocks and reinnervation with long-term IVIg. Neurology. 2004;63:1264–9. 15477549 10.1212/01.wnl.0000140497.85952.fa

[22] Vucic S, Cairns KD, Black KR, Cervical nerve root stimulation. Part I: technical aspects and normal data. Clin Neurophysiol. 2006;117:392–7. 16403485 10.1016/j.clinph.2005.10.011

[23] Vucic S, Black K, Chong PS, Multifocal motor neuropathy with conduction block: Distribution of demyelination and axonal degeneration. Clin Neurophysiol. 2007;118:124–30. 17095292 10.1016/j.clinph.2006.09.020

[24] Pestronk A, Choksi R, Multifocal motor neuropathy. Serum IgM anti-GM1 ganglioside antibodies in most patients detected using covalent linkage of GM1 to ELISA plates. Neurology. 1997;49:1289–92. 9371910 10.1212/wnl.49.5.1289

[25] Pestronk A, Multifocal motor neuropathy: diagnosis and treatment. Neurology. 1998;51:S22–4. 9851726 10.1212/wnl.51.6_suppl_5.s22

[26] Uncini A, Santoro M, Corbo M, Conduction abnormalities induced by sera of patients with multifocal motor neuropathy and anti-GM1 antibodies. Muscle Nerve. 1993;16:610–5. 8502258 10.1002/mus.880160606

[27] Kiernan MC, Guglielmi JM, Kaji R, Evidence for axonal membrane hyperpolarization in multifocal motor neuropathy with conduction block. Brain. 2002;125:664–75. 11872621 10.1093/brain/awf041

[28] European Federation of Neurological Societies/Peripheral Nerve Society guideline on management of multifocal motor neuropathy. Report of a joint task force of the European Federation of Neurological Societies and the Peripheral Nerve Society--first revision. J Peripher Nerv Syst. 2010;15:295–301. 21199100 10.1111/j.1529-8027.2010.00290.x

[29] van Schaik IN, Léger J-M Nobile-Orazio E. Multifocal Motor Neuropathy. In: Gilhus NE, Barnes MP and Brainin M (eds) European Handbook of Neurological Management, Blackwell Publishing Ltd, 2011.

[30] Willison HJ, Yuki N. Peripheral neuropathies and anti-glycolipid antibodies. Brain. 2002;125:2591–625. 12429589 10.1093/brain/awf272

[31] van Schaik IN, Bossuyt PM, Brand A, Diagnostic value of GM1 antibodies in motor neuron disorders and neuropathies: a meta-analysis. Neurology. 1995;45:1570–7. 7644057 10.1212/wnl.45.8.1570

[32] May C, Nordhoff E, Casjens S, Highly immunoreactive IgG antibodies directed against a set of twenty human proteins in the sera of patients with amyotrophic lateral sclerosis identified by protein array. PLoS One. 2014;9:e89596. 24586901 10.1371/journal.pone.0089596 PMC3935926

[33] Emilien D, Hugh W. Diagnostic utility of auto antibodies in inflammatory nerve disorders. J Neuromuscul Dis. 2015;2:107–12. 27858733 10.3233/JND-150078 PMC5271420

[34] van Schaik IN, Bossuyt PM, Brand A, Vermeulen M. Diagnostic value of GM1 antibodies in motor neuron disorders and neuropathies: a meta-analysis. Neurology. 1995;45:1570–7. 7644057 10.1212/wnl.45.8.1570

[35] Simon NG, Ayer G, Lomen-Hoerth C. Is IVIg therapy warranted in progressive lower motor neuron syndromes without conduction block? Neurology. 2013;81:2116–20. 24212395 10.1212/01.wnl.0000437301.28441.7e PMC3863347

[36] Van Asseldonk JT, Van den Berg LH, Van den Berg-Vos RM, Demyelination and axonal loss in multifocal motor neuropathy: distribution and relation to weakness. Brain. 2003;126:186–98. 12477706 10.1093/brain/awg019

[37] Menon P, Geevasinga N, Yiannikas C, Sensitivity and specificity of threshold tracking transcranial magnetic stimulation for diagnosis of amyotrophic lateral sclerosis: a prospective study. Lancet Neurol. 2015;14:478–84. 25843898 10.1016/S1474-4422(15)00014-9

[38] Rattay TW, Winter N, Decard BF, Nerve ultrasound as follow-up tool in treated multifocal motor neuropathy. Eur J Neurol. 2017;24:1125–34. 28681489 10.1111/ene.13344

[39] Grimm A, Vittore D, Schubert V, Ultrasound pattern sum score, homogeneity score and regional nerve enlargement index for differentiation of demyelinating inflammatory and hereditary neuropathies. Clin Neurophysiol. 2016;127:2618–24. 27291881 10.1016/j.clinph.2016.04.009

[40] Carvalho MD, Swash M. Awaji diagnostic algorithm increases sensitivity of El Escorial criteria for ALS diagnosis. Amyotroph Lateral Scler. 2009;10:53–7. 18985466 10.1080/17482960802521126

[41] Bouche P, Moulonguet A, Younes-Chennoufi AB, Multifocal motor neuropathy with conduction block: a study of 24 patients. J Neurol Neurosurg Psychiatry. 1995;59:38–44. 7608707 10.1136/jnnp.59.1.38 PMC1073599

[42] Bentes C, de Carvalho M, Evangelista T, Multifocal motor neuropathy mimicking motor neuron disease: nine cases. J Neurol Sci. 1999;169:76–9. 10540011 10.1016/s0022-510x(99)00219-1

[43] Turner MR, Talbot K, Mimics and chameleons in motor neurone disease. Pract Neurol. 2013;13:153–64. 23616620 10.1136/practneurol-2013-000557 PMC3664389

[44] European Federation of Neurological Societies/Peripheral Nerve Society Guideline on management of multifocal motor neuropathy. Report of a joint task force of the European Federation of Neurological Societies and the Peripheral Nerve Society. J Peripher Nerv Syst. 2006;11:1–8. 16519777 10.1111/j.1085-9489.2006.00058.x

[45] Nguyen TP, Chaudhry V. Multifocal motor neuropathy. Neurol India. 2011;59:700–6. 22019654 10.4103/0028-3886.86544

[46] Harbo T, Andersen H, Hess A, Subcutaneous versus intravenous immunoglobulin in multifocal motor neuropathy: a randomized, single-blinded cross-over trial. Eur J Neurol. 2009;16:631–8. 19236457 10.1111/j.1468-1331.2009.02568.x

[47] Dacci P, Riva N, Scarlato M, Subcutaneous immunoglobulin therapy for the treatment of multifocal motor neuropathy: a case report. Neurol Sci. 2010;31:829–31. 20574753 10.1007/s10072-010-0352-z

[48] Eftimov F, Vermeulen M, de Haan RJ, Subcutaneous immunoglobulin therapy for multifocal motor neuropathy. J Peripher Nerv Syst. 2009;14:93–100. 19691531 10.1111/j.1529-8027.2009.00218.x

[49] Misbah S, Sturzenegger MH, Borte M, Subcutaneous immunoglobulin: opportunities and outlook. Clin Exp Immunol. 2009;158 Suppl 1:51–9. 19883424 10.1111/j.1365-2249.2009.04027.x PMC2801034

[50] Umapathi T, Hughes RA, Nobile-Orazio E, Immunosuppressant and immunomodulatory treatments for multifocal motor neuropathy. Cochrane Database Syst Rev. 2009;CD003217. 19160219 10.1002/14651858.CD003217.pub3

[51] Carpo M, Cappellari A, Mora G, Deterioration of multifocal motor neuropathy after plasma exchange. Neurology. 1998;50:1480–2. 9596014 10.1212/wnl.50.5.1480

[52] Donaghy M, Mills KR, Boniface SJ, Pure motor demyelinating neuropathy: deterioration after steroid treatment and improvement with intravenous immunoglobulin. J Neurol Neurosurg Psychiatry. 1994;57:778–83. 8021660 10.1136/jnnp.57.7.778 PMC1073014

[53] Nemni R, Santuccio G, Calabrese E, Efficacy of cyclosporine treatment in multifocal motor neuropathy. J Neurol. 2003;250:1118–20. 14504978 10.1007/s00415-003-0131-3

[54] Chaudhry V, Cornblath DR. An open-label trial of rituximab (Rituxan^®^) in multifocal motor neuropathy. J Peripher Nerv Syst. 2010;15:196–201. 21040141 10.1111/j.1529-8027.2010.00270.x

[55] Rothstein JD, Edaravone: A new drug approved for ALS. Cell. 2017;171:725. 29100067 10.1016/j.cell.2017.10.011

[56] Writing Group; Edaravone (MCI-186) ALS 19 Study Group. Safety and efficacy of edaravone in well defined patients with amyotrophic lateral sclerosis: a randomised, double-blind, placebo-controlled trial. Lancet Neurol. 2017;16:505–12. 28522181 10.1016/S1474-4422(17)30115-1

[57] Furukawa T, Matsui N, Fujita K, CSF cytokine profile distinguishes multifocal motor neuropathy from progressive muscular atrophy. Neurol Neuroimmunol Neuroinflamm. 2015;2:e138. 26280014 10.1212/NXI.0000000000000138 PMC4529282

[58] Loewenbruck KF, Liesenberg J, Dittrich M, Nerve ultrasound in the differentiation of multifocal motor neuropathy (MMN) and amyotrophic lateral sclerosis with predominant lower motor neuron disease (ALS/LMND). J Neurol. 2016;263:35–44. 26477025 10.1007/s00415-015-7927-9

[59] Nodera H, Izumi Y, Takamatsu N, Cervical root sonography to differentiate multifocal motor neuropathy from ALS. J Med Invest. 2016;63:104–7. 27040062 10.2152/jmi.63.104

